# Conditional protein degradation in *Yarrowia lipolytica* using the auxin-inducible degron

**DOI:** 10.3389/fbioe.2023.1188119

**Published:** 2023-05-31

**Authors:** Zhenlin Han, Jessica Maruwan, Yinjie Tang, Wei Wen Su

**Affiliations:** ^1^ Department of Molecular Biosciences and Bioengineering, University of Hawai’i at Manoa, Honolulu, HI, United States; ^2^ Department of Energy, Environmental and Chemical Engineering, Washington University, Saint Louis, MO, United States

**Keywords:** conditional protein degradation, degron, metabolic engineering, synthetic biology, *Yarrowia lipolytica*

## Abstract

Conditional protein degradation is a powerful tool for controlled protein knockdown. The auxin-inducible degron (AID) technology uses a plant auxin to induce depletion of degron-tagged proteins, and it has been shown to be functional in several non-plant eukaryotes. In this study, we demonstrated AID-based protein knockdown in an industrially important oleaginous yeast *Yarrowia lipolytica*. Using the mini-IAA7 (mIAA7) degron derived from *Arabidopsis* IAA7, coupled with an *Oryza sativa* TIR1 (OsTIR1) plant auxin receptor F-box protein (expressed from the copper-inducible MT2 promoter), C-terminal degron-tagged superfolder GFP could be degraded in *Yarrowia lipolytica* upon addition of copper and the synthetic auxin 1-Naphthaleneacetic acid (NAA). However, leaky degradation of the degron-tagged GFP in the absence of NAA was also noted. This NAA-independent degradation was largely eliminated by replacing the wild-type OsTIR1 and NAA with the OsTIR1^F74A^ variant and the auxin derivative 5-Ad-IAA, respectively. Degradation of the degron-tagged GFP was rapid and efficient. However, Western blot analysis revealed cellular proteolytic cleavage within the mIAA7 degron sequence, leading to the production of a GFP sub-population lacking an intact degron. The utility of the mIAA7/OsTIR1^F74A^ system was further explored in controlled degradation of a metabolic enzyme, β-carotene ketolase, which converts β-carotene to canthaxanthin via echinenone. This enzyme was tagged with the mIAA7 degron and expressed in a β-carotene producing *Y. lipolytica* strain that also expressed OsTIR1^F74A^ controlled by the MT2 promoter. By adding copper and 5-Ad-IAA at the time of culture inoculation, canthaxanthin production was found to be reduced by about 50% on day five compared to the control culture without adding 5-Ad-IAA. This is the first report that demonstrates the efficacy of the AID system in *Y. lipolytica*. Further improvement of AID-based protein knockdown in *Y. lipolytica* may be achieved by preventing proteolytic removal of the mIAA7 degron tag.

## 1 Introduction

Degron-mediated protein degradation is an important tool for rewiring metabolic pathways, studying protein functions, and creating novel synthetic-biology systems (Natsume and Kanemaki, 2017). Auxin-inducible degron (AID) is a degron system originating from plants, but it has been successfully applied to various non-plant eukaryotic organisms including baker’s yeast *Saccharomyces cerevisiae,* mammalian cells, and transgenic mice (Nishimura et al., 2009; Yesbolatova et al., 2020). It has gained considerable interests for biotechnological applications owing to its specificity, degradation speed, and inducibility.

In plants, the Transport Inhibitor Response 1 (TIR1) auxin-receptor F-box protein, which is a component of the SCF (Skp1, Cullins, F-box proteins) multi-subunit E3 ubiquitin ligase complex, recruits the auxin-responsive proteins in the presence of indole-3-acetic acid (IAA), for ubiquitination and degradation. It was discovered by Nishimura et al. (2009) that the auxin-dependent protein degradation pathway could be transferred to *S. cerevisiae* and mammalian cells, while the heterologous AID system consists of three main components: an AID degron (a motif derived from the auxin-responsive proteins, such as *Arabidopsis thaliana* IAA17) fused to a target protein to be degraded, the TIR1 protein, and the auxin inducer molecule. It was reasoned that the heterologously expressed TIR1 protein can bind the host Skp1 adaptor protein to form a functional SCF ubiquitin ligase complex in the heterologous host, since the Skp1 protein is conserved in many species including yeast and humans (Zhang et al., 1995). Therefore, the plant’s F-box protein in theory should be able to interact with different species’ Skp1 protein, which means that AID system should be applicable to other eukaryotic species. Unfortunately, this might not be true in all cases. For example, Kanke et al. (2011) reported inefficient binding of *A. thaliana* TIR1 (AtTIR1) to endogenous Skp1 in fission yeast (*Schizosaccharomyces pombe*). Fusing *S. pombe* Skp1 to AtTIR1 was shown to increase the AID degradation efficiency in the fission yeast. In this case, the low affinity between AtTIR1 and fission yeast Skp1 is no longer an issue. However, when expressed from a strong promoter, the AtTIR1-Skp1 fusion protein was found to be toxic to *S. pombe* (Kanke et al., 2011).

The original AID degron system (AID1) has several other drawbacks as well, such as auxin independent degradation and requirement of high doses of auxin (Yamaguchi et al., 2019). More recently, an improved AID2 system was developed, which involves mutating Phe74 to either Ala or Gly (i.e., F74A or F74G) in *Oryza sativa* TIR1 (OsTIR1), and uses 5-Adamantyl-IAA (5-Ad-IAA) or 5-phenyl-IAA (5-Ph-IAA) as the inducer (Yesbolatova et al., 2020). The F74 A/G mutation in OsTIR1 would enlarge its auxin-binding pocket, so that the mutant shows a high binding affinity towards 5-Ad-IAA and 5-Ph-IAA, and a low affinity towards IAA (Nishimura et al., 2020; Zhang et al., 2022). As a result, the AID2 system is reported to have higher degradation efficiency, much lower leaky degradation, and require much lower auxin doses, and it has been shown to work in *S. cerevisiae,* chicken DT40 cells, and various vertebrate cell lines (Nishimura et al., 2020; Nishimura and Fukagawa, 2021; Watson et al., 2021; Zhang et al., 2022). However, the AID systems have shown varying degrees of effectiveness in different host organisms, and it has not been validated in the industrially important oleaginous yeast, *Y.lipolytica*, which is the focus of this study.

*Y. lipolytica* is an important non-model yeast widely considered as a promising industrial chassis for valorizing renewable carbon feedstocks to a wide variety of high-value chemicals (Niehus et al., 2018). As an oleaginous yeast, it is very efficient in *de novo* lipid biosynthesis, and can accumulate a high level of lipids in the cells using simple sugars as the carbon source. Therefore, *Y. lipolytica* is widely used for converting renewable sugar feedstocks into lipids as biofuel. It is also capable of utilizing lipids as the sole carbon source, and hence a promising microbial platform for valorizing renewable waste lipid feedstock (Li et al., 2020). Significant efforts have been made to engineer *Y. lipolytica* as a chassis organism for producing useful compounds. While some genetic tools are available for engineering *Y. lipolytica* metabolism, addition of the degron technology to the toolbox will significantly expand the possibilities of improving existing traits or creating novel traits in this important organism.

The objective of this study was to evaluate the auxin-inducible degron system in *Y. lipolytica*. Superfolder GFP (Pedelacq et al., 2006) tagged with the mini-IAA7 degron tag (Li et al., 2019) was used as a model protein to characterize the auxin-dependent degradation by co-expressing OsTIR1 vs. OsTIR1^F74A^, respectively. The degron system was further validated by studying auxin-dependent degradation of a metabolic enzyme, the β-carotene ketolase, and its effect on carotenoid production in an engineered *Y. lipolytica* strain. The present study is significant as it lays the foundation for applying the conditional-degron technology to create novel and improved *Y. lipolytica* microbial cell factories.

## 2 Materials and methods

### 2.1 Strains and chemicals

*Escherichia coli* strain DH5α was used for plasmid manipulation and propagation. *S. cerevisiae* strain EBY100 (Boder and Wittrup, 1997) was used for assembly of DNA fragments based on yeast recombination-based cloning. *Y. lipolytica* Po1g (Nicaud et al., 2002) and β-carotene producing *Y. lipolytica* strain ST6057 (Kildegaard et al., 2017) were used for protein expression and carotenoid production, respectively. Synthetic auxin derivatives 5-Ad-IAA and 5-Ph-IAA were ordered from TCI America (Portland, OR) and R&D Systems (Minneapolis, MN), respectively. All other chemicals are analytical grades from Sigma Aldrich (St. Louis, MO).

### 2.2 Plasmid construction

Plasmid pEHT-G, which targets the intE_4 locus for insertion of the GFP expression cassette and the hygromycin selection marker cassette, is constructed as follows. The TEF_intron_ promoter (Tai and Stephanopoulos, 2013) fragment (*PrTEFin*), which is used to drive the GFP expression, was amplified with the forward primer C1TIF and reverse primer TINR using the plasmid pCFB4666 (Addgene #106144) as template (Holkenbrink et al., 2018). The *GFP* fragment was amplified with the forward primer TIGF and reverse primer SGPR using the plasmid sfGFP-pBAD (Addgene #54519) as template. Then *PrTEFin* and *GFP* fragments were assembled into AsiSI-linearized pCfB5219 plasmid (Addgene #106135) using NEBuilder HiFi DNA assembly (New England Biolabs, Ipswich, MA).

To construct plasmid pEHT-GI for expressing the GFP-mIAA7 fusion protein, the *GFP* fragment was amplified with the forward primer C1TIF and reverse primer SFGR using the plasmid sfGFP-pBAD as template. The *mIAA7* fragment was obtained with the forward primer IA7F and reverse primer IA7LR using the *Yarrowia* codon-optimized *mIAA7* fragment (synthesized by Twist Bioscience, San Francisco, CA) as template. The two fragments were then assembled into AsiSI-linearized pCfB5219 plasmid using NEBuilder HiFi DNA assembly.

To construct pEHT-GIWH, the *Wps-H6* fragment encoding the β-carotene ketolase from *Paracoccus* sp. N81106 was obtained with the forward primer A7WF and reverse primer PWH6R using the genomic DNA of ST7403 (Kildegaard et al., 2017) as template to introduce a hexa-His (H6) tag at the C-terminus of Wps. Subsequently, the *Wps-H6* fragment was assembled into SalI-linearized pEHT-GI using NEBuilder HiFi DNA assembly. To construct pEHT-WH, the *Wps-H6* fragment was amplified from the ST7403 genomic DNA using the forward primer TWPF and reverse primer PWH6R. The resulting product *Wps-H6* was then joined with *PrTEFin* and assembled into AsiSI-linearized plasmid pCfB5219 using NEBuilder HiFi DNA assembly.

To construct plasmids for integrating the OsTIR1 or OsTIR1^F74A^ expression cassette at the int_F2 locus, plasmid pFLM-G was first constructed by double digestion of pCU-IntF2U-LoxP-Leu2-LoxP-hp4D-XPR2-IntF2D (Li et al., 2020) with SalI and BamHI to remove the hp4D prompter fragment and assemble with the *MT2 promoter* (Xiong and Chen, 2020) and *GFP* fragments using NEBuilder HiFi DNA assembly. The *MT2 promoter* fragment was amplified using the forward primer MT2F and reverse primer MT2R, from the Po1g genomic DNA. The *GFP* fragment was amplified with the forward primer T2GF and reverse primer SFGN using plasmid sfGFP-pBAD as the template. The *OsTIR1* fragment was amplified with the forward primer MOSF and reverse primer MOSR, using a synthetic codon-optimized *OsTIR1* sequence as template. To obtain the F74A mutant of *OsTIR1*, the forward primer F74AF and reverse primer F74AR were used. Finally, *OsTIR1* and *OsTIR1* ^*F74A*^ were assembled into BamHI/NheI linearized pFLM-G with NEBuilder HiFi DNA assembly to produce pFLM-OsTIR1 and pFLM-F74A, respectively. All primers used in this study are listed in Supplementary Table S1. Detailed plasmid maps and linear DNA structures of major constructs used in this study are presented in Supplementary Figures S1, S2.

### 2.3 Recombinant *Yarrowia* strain development

Plasmids pEHT-G and pEHT-GI were linearized with NotI and transformed into Po1g, and pFLM-G, and pEHT-GIWH were linearized with NotI and transformed into ST6057 using the lithium acetate (LiAc) method (Marsafari and Xu, 2020), with hygromycin B selection (250 μg/mL) on YPD plates. Resulting colonies were screened based on GFP fluorescence and further confirmed by PCR. Colony GFP fluorescence was visualized using the Dark Reader blue transilluminator (Clare Chemical Research, Dolores, CO). Plasmids pFLM-OsTIR1 and pFLM-F74A were linearized with NotI and NruI and then transformed into Po1g-EHT-GI, respectively. pFLM-F74A was also transformed into ST6057-EHT-GIWH and transformants selected using leucine dropout media. The resulting strains were screened with PCR, using genomic DNA as template. Major *Y. lipolytica* strains used in this study are summarized in Table 1.

**TABLE 1:** Major *Y. lipolytica* strains used in this study.

Strain name	Gene cassettes expressed^a^	Host
GI	*PrTEFin-sfGFP-mIAA7-TPex20*	Po1g
GI/WT	*PrTEFin-sfGFP-mIAA7-TPex20/PrMT2-OsTIR1-TXpr2*	Po1g
GI/F74A	*PrTEFin-sfGFP-mIAA7-TPex20/PrMT2-OsTIR1* ^*F74A*^ *-TXpr2*	Po1g
G	*PrTEFin-sfGFP-TPex20*	Po1g
MG	*PrMT2-sfGFP-TXpr2*	ST6057
GIWH	*PrTEFin-sfGFP-mIAA7-Wps-H6-TPex20*	ST6057
GIWH/F74A	*PrTEFin-sfGFP-mIAA7-Wps-H6-TPex20/PrMT2-OsTIR1* ^*F74A*^ *-TXpr2*	ST6057
WH	*PrTEFin-Wps-H6-TPex20*	ST6057

^a^ *PrTEFin*, TEF_intron_ promoter; *PrMT2*, MT2 promoter; *TPex20*, Pex20 terminator; *TXpr2*, Xpr2 terminator; *sfGFP*, *mIAA7*, *Wps*, and *H6*, genes encoding sfGFP, mIAA7, Wps, and hexa-His tag, respectively.

### 2.4 Culture conditions for evaluating AID efficacy in *Y. lipolytica*

Po1g-GI/WT and Po1g-GI/F74A were grown at 28°C in 20 mL of YPD medium in a 250-mL baffled flask for 24 h, respectively, then CuSO_4_ was added to a final concentration as specified. Twelve hours later, 1-Naphthaleneacetic acid (NAA) or 5-Ad-IAA (or 5-Ph-IAA, as specified) was added into Po1g-GI/WT and Po1g-GI/F74A cultures to a final concentration of 0.5 mM and 1 μM, respectively. The GFP fluorescence intensity and optical density of the culture were measured every hour to monitor the degradation of the sfGFP-mIAA7 fusion protein based on the GFP fluorescence and Western blot analysis. To evaluate the AID efficacy in regulating carotenoid biosynthesis in *Y. lipolytica*, ST6057-GIWH/F74A was cultured in YPD with 0.2 mM CuSO_4_ and 1 µM 5-Ad-IAA added at the time of inoculation. All culture experiments were conducted in shake flasks.

### 2.5 Western blot analysis and GFP culture fluorescence measurement

All culture samples were centrifuged and cell pellets rinsed three times with PBS buffer and stored at −80°C for subsequent Western blot and/or culture GFP fluorescence analysis. Each cell pellet sample was thawed, resuspended in PBS buffer, and diluted to OD_600_ = 0.3 to measure the culture GFP fluorescence with a Hitachi F-2500 fluorescence spectrophotometer. To prepare protein extracts for Western blot analysis, cell pellets were resuspended in 10% TCA buffer, followed by homogenization using Mini-Beadbeater-16 (Biospec model 607, Bartlesville, OK) with zirconia/silica beads (0.5 mm) in 3 × 1 min bursts. The extracted protein pellet was resuspended in a resuspension buffer as described previously (Cox et al., 1997). The protein concentration in the extract was measured using the Nanodrop ND-1000 spectrophotometer. The protein extract is then subject to SDS-PAGE and Western blot analysis. SDS-PAGE was performed using the 12% polyacrylamide gel; about 5 µg of total soluble protein for each sample was mixed with 5× loading buffer and subjected to electrophoresis. Proteins separated in SDS-PAGE gel were electroblotted onto a polyvinylidene difluoride (PVDF) membrane, and probed with anti-GFP antibody or anti-His Tag antibody (Genscript, Piscataway NJ), as described previously (Zhang et al., 2017).

### 2.6 qRT-PCR

ST6057-GI/F74A and ST6057-MG, respectively, was grown at 28°C in the YPD medium for 24 h, and CuSO_4_ was then added into the culture at a final concentration of 0.2 mM. Cell culture samples were taken every 30 min for 3 h, and on the 4^th^ hour. During each sampling, the cells were rinsed with sterile water, and the cell pellets were stored at −80°C. After all samples were collected, RNA was extracted from each sample with Quick Fungal Bacterial MiniPrep kit (ZymoResearch). The quality and concentration of RNA were assessed with Nanodrop ND-1000. Then RT reaction was conducted with 600 ng of RNA for each sample, using the LunaScript^®^ RT SuperMix Kit (NEB). The resulting cDNA product was diluted 25-fold and used for qPCR with SYBR green dye. Primer sets Q74F1/Q74R1 and QSGF/QSGR were used in qPCR to detect *OsTIR1* ^*F74A*^ and *GFP* transcripts, respectively. Primer sets QACT1F/QACT1R and QTEF1F/QTEF1R were used to detect transcript levels of ACT1 and TEF1 reference genes, respectively.

### 2.7 MT2 promoter-GFP fluorescence time course upon copper induction

ST6057-MG was grown at 28°C in the YPD medium for 24 h, then CuSO_4_ was added into the culture at a final concentration of 0.2 mM. Cell culture samples were taken every 30 min for 3 h, and on the 4^th^ hour. During each sampling, the cells were rinsed and resuspended in 200 µL of sterile water. Samples were diluted 20-fold and loaded into a 96-well plate in triplicates. Cell OD_600_ and culture GFP fluorescence were then measured with the Tecan Infinite M Plex plate reader. GFP fluorescence was measured with the excitation wavelength of 470 nm, and emission wavelength of 511 nm.

### 2.8 High-performance liquid chromatography (HPLC) analysis of carotenoids

ST6057-GIWH/F74A was grown at 28°C in the YPD medium. Three cultures were set up for comparison. At the time of inoculation, one culture was supplemented with CuSO_4_ (0.2 mM), one with both CuSO_4_ (0.2 mM) and 5-Ad-IAA (1 µM), and the third is a control, without supplementation of CuSO_4_ or auxin. Samples were taken during the course of the culture to monitor the carotenoid production. The carotenoids in the cell samples were separated and quantified using HPLC as described previously (Li et al., 2020). The titer of the major carotenoid product, canthaxanthin, was estimated based on the calibration curve with a canthaxanthin standard (Sigma Aldrich 32993).

## 3 Results

### 3.1 Protein degradation using mIAA7-degron paired with OsTIR1/NAA vs. OsTIR1^F74A^/5-Ad-IAA

**FIGURE 1:**
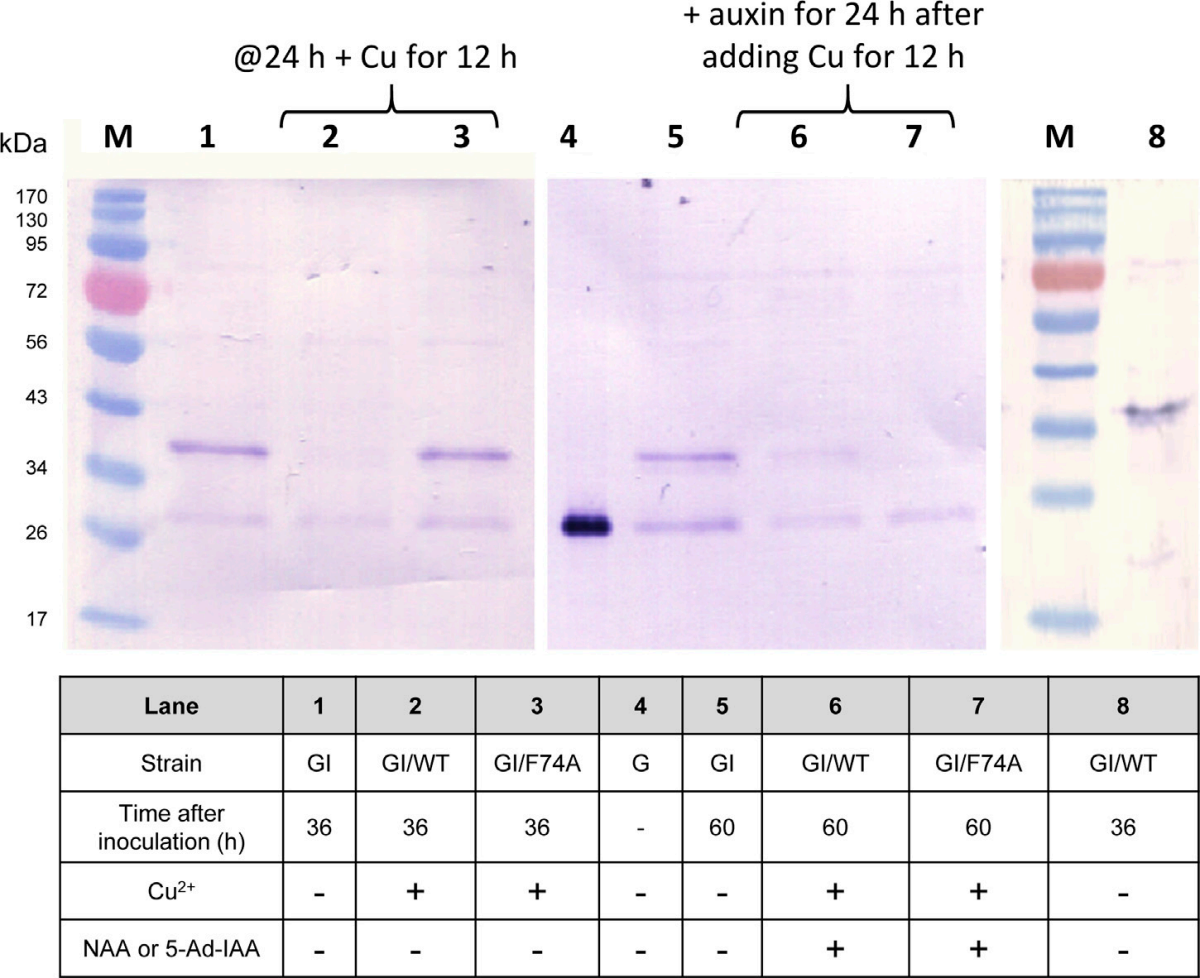
GFP Western blot analysis of protein degradation using the mIAA7-degron paired with OsTIR1/NAA and OsTIR1^F74A^/5-Ad-IAA, respectively. Refer to text for further experimental detail.

To examine whether the AID system is functional in *Y. lipolytica*, a GFP reporter tagged with a C-terminal mIAA7 degron tag was expressed alone, or co-expressed with either the wild type OsTIR1 or its F74A variant (OsTIR1^F74A^). We used superfolder GFP as the reporter instead of the enhanced GFP (EGFP) commonly used in *S. cerevisiae* because the latter was found in our prior studies to be inactive when expressed in *Y. lipolytica*. The mIAA7 degron is composed of amino acids 37–104 from the *A. thaliana* IAA7 protein (an auxin/IAA response transcription repressor). OsTIR1 is a plant auxin receptor F-box protein. Both OsTIR1 and OsTIR1^F74A^ genes were codon-optimized for *Y. lipolytica* expression. The expression of the GFP-mIAA7 fusion protein was driven by the TEF_intron_ promoter, whereas OsTIR1 (or OsTIR1^F74A^) was expressed from the copper-inducible MT2 promoter (Xiong and Chen, 2020). NAA at 0.5 mM and 5-Ad-IAA at 1 µM were used with OsTIR1 and OsTIR1^F74A^, respectively. Three *Y. lipolytica* strains GI, GI/WT, and GI/F74A were compared. The GI culture (which serves as a control) was inoculated into the YPD medium without adding additional chemicals. The GI/WT culture was inoculated into the YPD medium and cultured for 24 h, followed by supplementing with 0.2 mM of copper sulfate, and 12 h later 0.5 mM of NAA was added. The GI/F74A culture was treated similarly as the GI/WT culture, except that 0.5 mM NAA was replaced with 1 µM of 5-Ad-IAA. For all three cultures, samples were taken 36 h and 60 h post inoculation for Western blot analysis using an anti-GFP antibody. The result is presented in Figure 1. All samples in the Western blot were loaded based on the same total soluble protein concentration. For the GI control culture, besides the full-length fusion protein (with a molecular mass of 34.4 kDa), another immunoreactive band with a molecular mass similar to GFP was also detected on the Western blot. The proportion of this lower band grew overtime between 36 and 60 h post inoculation (cf. lanes 1 and 5 in Figure 1). This result suggests that the C-terminal mIAA7 tag may be susceptible to intracellular proteolytic cleavage in *Y. lipolytica*. For the GI/WT culture, the GFP-mIAA7 fusion protein was found to be degraded even with only Cu^2+^ (but without NAA) added (cf. lanes 2 and 8, Figure 1), and a very small amount of the fusion protein was detected 24 h after adding NAA (lane 6, Figure 1). This result indicated leaky degradation and inefficient NAA-specific protein degradation for the wildtype OsTIR1/NAA system. This finding was further corroborated by comparing the GFP fluorescence of the GI/WT culture samples which shows a decrease in culture GFP fluorescence after adding copper but no further drop in GFP fluorescence was noted after adding NAA (data not shown). Unlike wildtype OsTIR1, the F74A variant of OsTIR1 provided highly efficient degradation of GFP-mIAA7 when induced using the synthetic IAA derivative 5-Ad-IAA, and showed little signs of leaky degradation. This can be seen clearly on Figure 1 by comparing lanes 3 and 7 that shows highly efficient 5-Ad-IAA-induced degradation of GFP-mIAA7, while little or no leaky degradation was detected in the absence of 5-Ad-IAA by comparing lanes 1 and 3.

### 3.2 Effect of copper concentration on the mIAA7/OsTIR1^F74A^/5-Ad-IAA AID system

**FIGURE 2:**
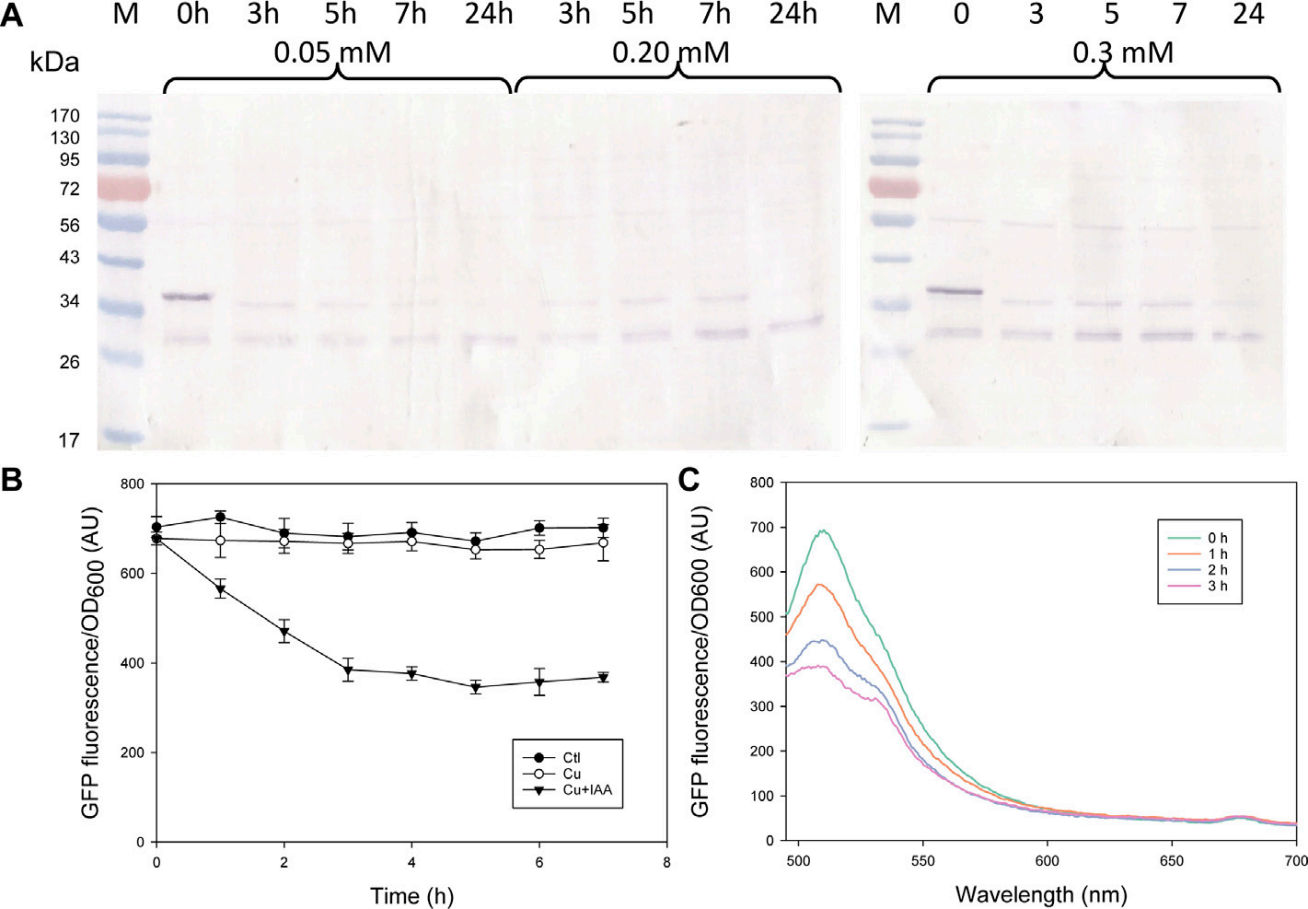
Characterization of the mIAA7/OsTIR1^F74A^/5-Ad-IAA AID system: effect of Cu^2+^ induction concentrations (0.05, 0.2, vs. 0.3 mM) and degradation time course of GFP-mIAA7 in *Y. lipolytica*. **(A)** GFP Western blot analysis of samples taken at 0, 3, 5, 7, and 24 h upon addition of 1 µM of 5-Ad-IAA. GI/F74A culture was grown for 24 h, followed by Cu^2+^ (0.2 mM) induction (to express OsTIR1^F74A^), and then 5-Ad-IAA was added 12 h later. **(B)** Time courses of GFP culture fluorescence upon adding Cu^2+^ (0.2 mM) alone or Cu^2+^ plus 5-Ad-IAA. **(C)** Changes in the GFP fluorescence spectra in response to addition of Cu^2+^ (0.2 mM) plus 5-Ad-IAA.

In designing the AID systems for this study, we controlled OsTIR1 and OsTIR1^F74A^ expression using the Cu^2+^-inducible MT2 promoter. By tuning the copper induction condition to control the level of OsTIR1 or OsTIR1^F74A^, it may help to minimize leaky protein degradation in the absence of exogenous auxin addition. In the case of OsTIR1^F74A^, we showed in Figure 1 that no leaky degradation was noted upon MT2 promoter induction using copper sulfate at 0.2 mM. However, the concentration of OsTIR1^F74A^ relative to the concentration of the degron-tagged protein may affect the efficiency of the AID mediated protein degradation. We therefore investigated the effect of copper induction concentration on the extent of AID-mediated GFP-mIAA7 degradation. Three copper inducer concentrations were tested (0.05, 0.2, and 0.3 mM). The GI/F74A culture was grown in YPD for 24 h, then Cu^2+^ was added at 0.05, 0.2, and 0.3 mM, respectively. At 36 h post inoculation, 1 µM of 5-Ad-IAA was added and the cultures were monitored at 0, 3, 5, 7, and 24 h post 5-Ad-IAA induction. The samples were analyzed using GFP Western blot (Figure 2A), from which, protein degradation was found to occur very rapidly (cf. 0 and 3 h samples), yet similar levels of degradation were noted at all three Cu^2+^ concentration tested. Given that the culture was induced by Cu^2+^ for 12 h before adding 5-Ad-IAA, sufficient OsTIR1^F74A^ might have already been accumulated even at the lowest Cu^2+^ induction concentration and thus was not limiting. After 5-Ad-IAA induction, nearly all full-length GFP-mIAA7 was depleted within 3 h regardless the Cu^2+^ concentration used to induce the MT2 promoter. Interestingly, the faint upper band detected in the samples at 3, 5, and 7 h post 5-Ad-IAA induction showed a slightly lower molecular mass than the full-length GFP-mIAA7 detected in the 0-h sample. This faint upper band was almost completely depleted at 24 h post IAA addition for all three Cu^2+^ concentrations tested. All samples contain a lower band (likely GFP) similar to that noted in Figure 1. The GI/F74A cultivation was repeated using 0.2 mM Cu^2+^ and 1 µM 5-Ad-IAA, and GFP culture fluorescence was monitored hourly for up to 7 h (Figure 2B). The culture fluorescence data (per culture OD) corroborate the Western blot result, and further inform the gradual degradation of GFP-mIAA7 during the first 3 h post 5-Ad-IAA induction, while the control and Cu^2+^-only culture displayed essentially constant fluorescence throughout the duration of the test. The residual background fluorescence seen in Figure 2B resulted mainly from culture autofluorescence and to a less extent the GFP cleaved from GFP-mIAA7 that was not degraded by OsTIR1^F74A^. As indicated in the fluorescence spectra of the culture samples (Figure 2C), the characteristic GFP emission peak occurring at around 510 nm subsided after 5-Ad-IAA addition, and essentially disappeared after 3 h.

**FIGURE 3:**
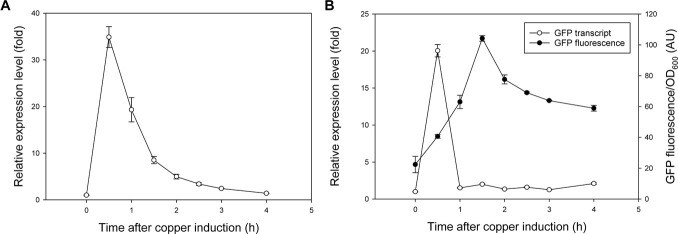
Induction kinetics of the MT2 promoter upon Cu^2+^ (0.2 mM) addition. **(A)** Transcript of OsTIR1^F74A^ expressed from the MT2 promoter (GI/F74A culture). **(B)** GFP expression driven by the MT2 promoter (MG culture), indicated by the GFP fluorescence and transcript time courses.

Because GFP-mIAA7 was driven by the constitutive TEF_intron_ promoter, whereas OsTIR1^F74A^ was by the copper-inducible MT2 promoter, we tracked the *OsTIR1* ^*F74A*^ transcript over a period of 4 h from the time of copper induction to inform the efficacy of the MT2 promoter (Figure 3A). The induction was found to be very rapid, and about 30∼40-fold increase in the *OsTIR1* ^*F74A*^ transcript was detected within 30 min after adding Cu^2+^ (0.2 mM), but the transcript level subsided quickly afterwards. We then examined another *Y. lipolytica* strain that expresses GFP driven by the MT2 promoter, by measuring time courses of GFP transcript and GFP fluorescence (indicating GFP protein concentrations) upon copper induction. As seen in Figure 3B, the *GFP* gene induction kinetics resembled that of the *OsTIR1* ^*F74A*^ gene (also driven by the MT2 promoter) in Figure 3A. Importantly, even though the *GFP* transcript quickly decreased to near the pre-induction level after it peaked, GFP protein accumulated above the pre-induction level. The OsTIR1^F74A^ protein level was not monitored, yet based on the data for GFP, and the fact that auxin-dependent GFP-mIAA7 degradation was found to be effective (Figures 1, 2), it is likely that the level of OsTIR1^F74A^ protein present in the *Y. lipolytica* cells was sufficient to enable efficient protein degradation.

### 3.3 Effect of auxin inducers on the mIAA7/OsTIR1^F74A^ AID system

**FIGURE 4:**
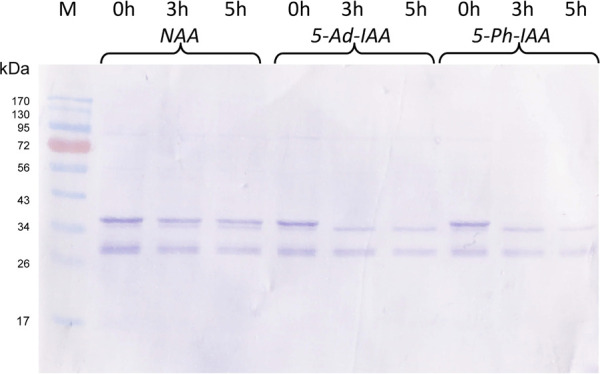
Characterization of the mIAA7/OsTIR1^F74A^ AID system: effect of different auxins. GFP Western blot analysis of samples taken at 0, 3, and 5 h upon addition of 1 µM of 5-Ad-IAA or 5-Ph-IAA, or 0.5 mM of NAA. The GI/F74A culture was grown for 24 h, followed by Cu^2+^ (0.2 mM) induction (to express OsTIR1^F74A^), and then the auxin was added 12 h later.

Besides 5-Ad-IAA, use of 5-Ph-IAA with OsTIR1^F74A^ was also reported in several studies. To compare the effect of different auxin inducers on protein degradation, Po1g-GI/F74A was grown at 28°C in the YPD medium for 24 h, CuSO_4_ added to a final concentration of 0.2 mM and further incubated for 12 more hours. NAA, 5-Ad-IAA, and 5-Ph-IAA were then supplemented at a final concentration of 0.5 mM, 1 μM, and 1 μM, respectively, and GFP-mIAA7 protein degradation was monitored using Western blot for up to 5 hours. As shown in Figure 4, 5-Ad-IAA and 5-Ph-IAA are equally effective in degrading the GFP-mIAA7. At 3 h post auxin induction, the full-length fusion protein was completely depleted, noting that the faint upper band seen under 3 and 5 h has a lower molecular mass than that of the full-length GFP-mIAA7 (as in Figure 2A). NAA on the other hand, despite at a much higher concentration, caused very modest protein degradation.

### 3.4 Conditional degradation of a biosynthetic enzyme using the mIAA7/OsTIR1^F74A^/5-Ad-IAA AID system

**FIGURE 5:**
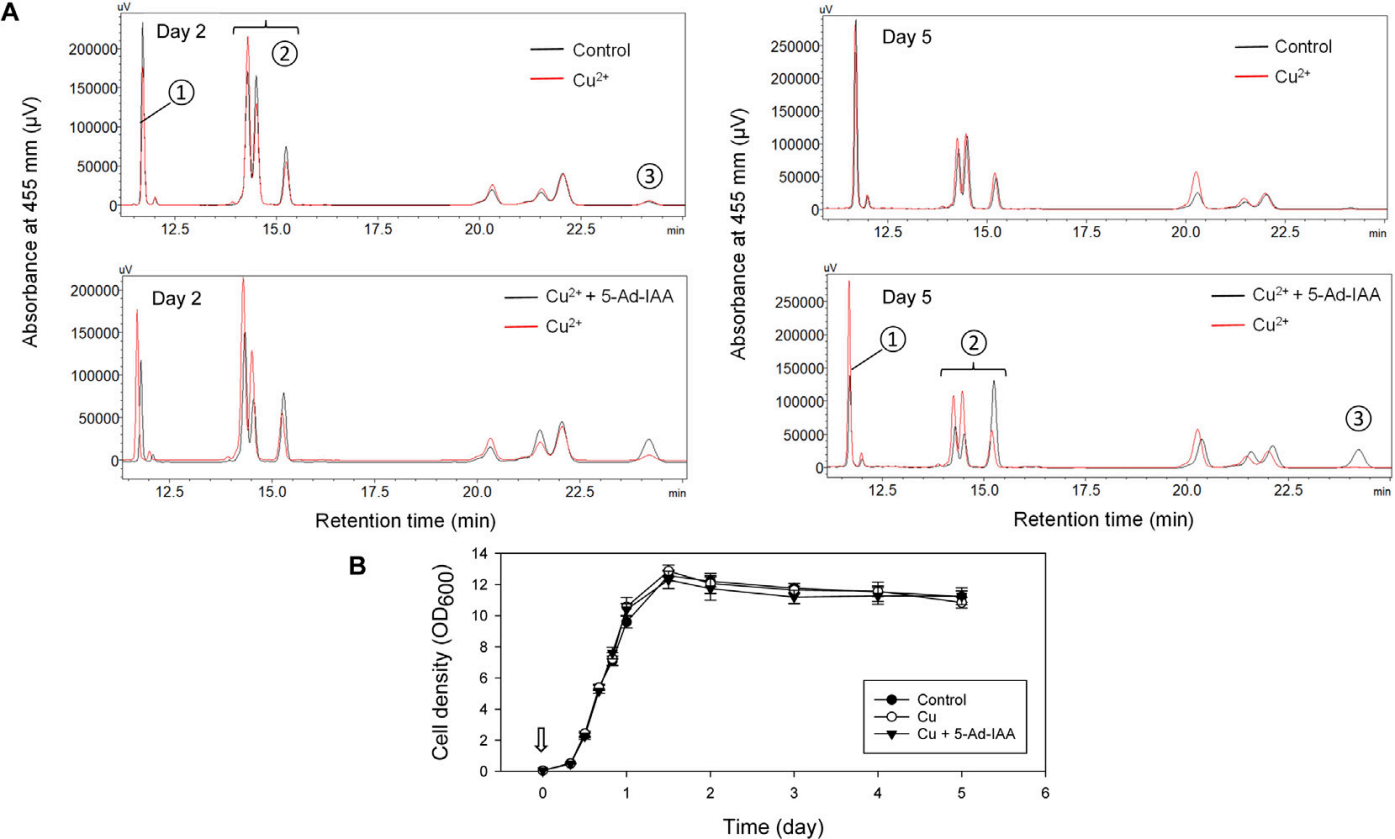
Controlling carotenoid biosynthesis in *Y. lipolytica* using the mIAA7/OsTIR1^F74A^ AID system as shown in HPLC analysis. Cu^2+^ (0.2 mM) and 5-Ad-IAA (1 µM) or Cu^2+^ alone was introduced at inoculation to the GIWH/F74A culture, and allowed to grow for 5 days. Control culture received no Cu^2+^ or 5-Ad-IAA. **(A)** HPLC analysis of samples taken on day 2 (left panel) and day 5 (right panel). Carotenoid species are labeled as follows: ① canthaxanthin, ② echinenone variants, and ③ β-carotene. **(B)** The corresponding growth curves (the arrow indicates the time Cu^2+^ and 5-Ad-IAA were added to the culture).

The mIAA7/OsTIR1^F74A^ degron system was further validated by examining auxin-dependent degradation of β-carotene ketolase, and its effect on carotenoid production in an engineered *Y. lipolytica* strain. The host *Y. lipolytica* strain ST6057 was engineered to produce β-carotene (Kildegaard et al., 2017), and it was further engineered in this study to create ST6057-GIWH/F74A that overexpresses the β-carotene ketolase Wps to convert β-carotene to canthaxanthin. Three culture treatments were set up and compared. Cu^2+^ (0.2 mM) and 5-Ad-IAA (1 µM) or Cu^2+^ alone was introduced at inoculation, vs. a control culture without supplementing Cu^2+^ or 5-Ad-IAA. Each culture was allowed to grow for 5 days. On day 2 and day 5, cells were extracted for carotenoid analysis using HPLC, and the resulting chromatograms (based on the same amount of cell biomass extracted across all three culture treatments) are presented in Figure 5. In our previous study, we identified the carotenoids produced by the *Y. lipolytica* ST7403 strain (which was derived from ST6057 by overexpressing β-carotene ketolase and β-carotene hydroxylase) using triple quadrupole LC/MS and published mass to charge ratio (m/z) of known carotenoid species (Li et al., 2020). In Figure 5, major carotenoid species appeared in the HPLC chromatograms were identified as follows: 1) canthaxanthin (retention time: 11.5–12 min), 2) echinenone variants (retention time: 14–15.5 min), and 3) β-carotene (retention time: 24–24.5 min). The canthaxanthin titer in the culture received both Cu^2+^ and 5-Ad-IAA (5.32 ± 0.25 mg/L on day 2 and 6.39 ± 0.43 on day 5) was about half of that seen in the control (10.62 ± 0.39 on day 2, and 13.61 ± 0.87 mg/L on day 5) or the culture receiving Cu^2+^ alone (8.12 ± 0.26 mg/L on day 2 and 13.57 ± 0.77 mg/L on day 5). Before β-carotene was converted to canthaxanthin, it was first transformed into echinenone variants. On day 5, in the culture treated with Cu^2+^ and 5-Ad-IAA, the early echinenone variant (appeared between 15 and 15.5 min) accumulated to a level that was about twice as much as those seen in the other two cultures, whereas the later-stage echinenone variants (between 14 and 15 min) accumulated to a level that was about half of those seen in the other two cultures. Furthermore, β-carotene accumulation was detected only in the culture received Cu^2+^ and 5-Ad-IAA due to lower Wps activity that reduced further conversion of β-carotene. All of these data indicate that the mIAA7/OsTIR1^F74A^ degron system rendered a lower Wps activity by lowering its abundance in the cells via protein degradation. Residual ketolase activities however persisted, and led to the formation of canthaxanthin and echinenone variants. In a related test, Cu^2+^ and 5-Ad-IAA were added 1 day after cell inoculation, and a similar carotenoid product trend emerged, i.e., lower canthaxanthin/later-stage echinenone and higher β-carotene/early-stage echinenone (data not shown). Addition of Cu^2+^ alone had almost no effect on the carotenoid production compared to control, indicating no IAA-independent leaky protein degradation with the mIAA7/OsTIR1^F74A^ degron system.

**FIGURE 6:**
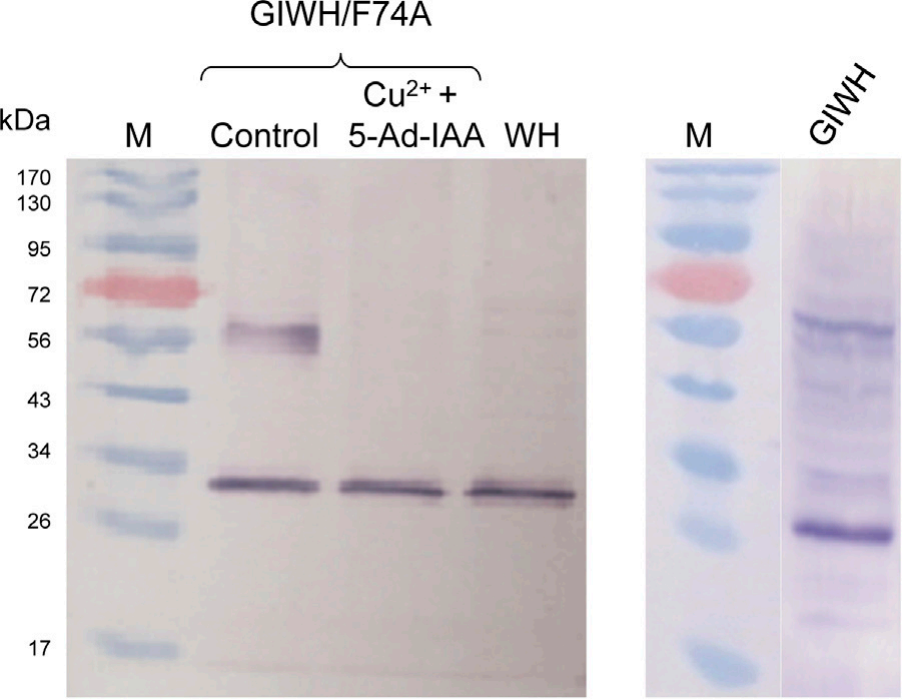
Western blot analysis of conditional degradation of β-carotene ketolase (Wps) using the mIAA7/OsTIR1^F74A^ AID system. Cu^2+^ (0.2 mM) and 5-Ad-IAA (1 µM) were introduced at inoculation to the GIWH/F74A culture and allowed to grow for 5 days before the cells were extracted for Western blot analysis using an anti-His-tag antibody (left panel). Control and WH cultures received no Cu^2+^ or 5-Ad-IAA. In a separate experiment, the GIWH culture (without co-expressing OsTIR1^F74A^) was grown for 1 day, before the cells were extracted for Western blot analysis using an anti-GFP antibody (right panel).

To further investigate the system at the protein level, Western blot analysis was conducted (Figure 6). The theoretical molecular mass of GFP-mIAA7-Wps-H6 and Wps-H6 are 61.8 kDa and 27.8 kDa, respectively. From the anti-His-tag Western blot in Figure 6 (left panel), the control culture showed an upper band corresponding to the full-length fusion protein and a lower band with a size similar to that of Wps, but the culture with copper plus 5-Ad-IAA only showed the lower band, indicating essentially complete degradation of the degron-tagged full-length fusion protein in the cells. However, the cleaved Wps product in the cells might contain no or only partial mIAA7 sequence which was insufficient for binding with OsTIR1^F74A^ and hence could not be degraded. The residual Wps was thus still able to convert β-carotene into downstream carotenoid products, and this may account for the canthaxanthin and echinenone variants seen in the HPLC chromatograms. In a separate experiment, *Y. lipolytica* strain ST6057-GIWH (without co-expressing OsTIR1^F74A^) was grown for 1 day, before the cells were extracted for Western blot analysis using an anti-GFP antibody (Figure 6, right panel). A major cleaved product with size similar to GFP is clearly visible on the blot along with the full-length fusion protein. Multiple faint immunoreactive bands are also visible with sizes between those of the full-length protein and the cleaved GFP protein. This result indicates that proteolytic cleavage within the mIAA7 sequence noted above with the GFP-mIAA7 protein also occurred in the GFP-mIAA7-Wps-H6 protein, despite that the mIAA7 sequence is not present at the protein termini. The mIAA7 peptide consists of the conserved F-box protein binding motif flanked by disordered sequences. It is known that long and unstructured peptide linkers may be prone to proteolytic digestion (Chen et al., 2013). Therefore, following initial intracellular proteolytic cleavage within the mIAA7 sequence, the remnant mIAA7 peptides may be further digested by carboxyl and/or amino-peptidases, leading to the formation of the GFP and Wps-H6 products seen on the western blots (Figure 6).

## 4 Discussion

Since Nishimura et al. (2009) reported that the plant auxin-dependent protein degradation pathway could be transferred to non-plant eukaryotic cells, the AID system has been applied to several different hosts, yet its utility in *Y. lipolytica* had not been demonstrated prior to the present study. In this study, we choose to focus on the mIAA7 degron, instead of the more commonly used mAID or AID* (a shorter version of mAID) degrons. In *Caenorhabditis elegans*, higher protein degradation efficiency was seen with mIAA7 than with AID* (Sepers et al., 2022). Though derived from different *A. thaliana* IAA response transcription repressor proteins (IAA7 vs. IAA17), and differ in primary sequences, both mIAA7 (IAA7 37-104) and AID* (IAA17 71-114) degrons contain the conserved domain II F-box protein binding motif, yet the former has a longer amino-terminal extension (which was reported to be important to TIR1-mediated protein degradation in plants) and contains no putative ubiquitination sites (and hence the degron tag itself is not ubiquitinated) (Sepers et al., 2022). The mIAA7 degron was first reported by Li et al. (2019) and was shown to be an optimal degron to pair with the *A. thaliana* AFB2 F-box protein to enable protein degradation. Sepers et al. (2022) used mIAA7 in combination with AtOsTIR1^F79G^ in their AID system. In the present study, we showed that mIAA7 worked well in combination with OsTIR1^F74A^ and either 5-Ad-IAA or 5-Ph-IAA in inducing rapid auxin-dependent protein degradation in *Y. lipolytica*. Importantly, the mIAA7/OsTIR1^F74A^/5-Ad-IAA degron system was shown in this study to be able to degrade both cytosolic (GFP) and integral membrane (Wps) proteins.

Whether it was fused internally or to the carboxyl terminus of the target protein, the mIAA7 degron was shown in this study to direct protein degradation in *Y. lipolytica*. However, Western blot analysis revealed cellular proteolytic cleavage within the mIAA7 degron sequence, leading to the production of target-protein subpopulations lacking an intact degron, which prevented complete degradation of the target protein upon auxin addition. By resolving the *A. thaliana* TIR1-auxin-IAA7 complex topology, it was shown that regions in the vicinity of the mostly conserved degron (VGWPP-[VI]-[RG]-x (2)-R) motif of IAA proteins are intrinsically disordered and they cooperatively position IAA protein on TIR1 (Niemeyer et al., 2020). The mIAA7 degron tag is only a portion of the IAA7 protein, and it consists of the conserved degron motif flanked by disordered sequences. The mIAA7 tag when fused between two other protein moieties or to the protein termini would likely be present as a highly disordered linker especially in the absence of the auxin inducer. Such a long (68 residues, 7.53 kDa) and disordered linker may be very prone to proteolytic digestion (Chen et al., 2013), which may explain why a portion of the degron-tagged proteins appeared to lose their degron tag, as shown in the Western blot results.

To reduce leaky degradation, in this study, the TIR1 expression was under the regulation of the copper-inducible MT2 promoter. As shown in Figure 3, MT2 promoter induction was very rapid, yet the transcript level subsided quickly after peaking at about 30 min after induction. The sharp decline in transcript level could be due to the copper (II) detoxification mechanism (Peng et al., 2015). Copper is an essential trace element yet it becomes toxic if not properly regulated. In the yeast *S. cerevisiae*, this mechanism entails reduction of copper (II) to copper (I), which is then bound to metallothionein or converted to copper metal, and may result in depletion of the cellular copper (II) inducer pool (Hassett and Kosman, 1995). A similar copper detoxification mechanism also exists in *Y. lipolytica* (Ran et al., 2023). Besides the copper-inducible promoter systems, alternative promoters (Sun et al., 2022) inducible by erythritol (Trassaert et al., 2017), xylose (Wei et al., 2020), and oleic acid (Sassi et al., 2016), respectively, may be considered for driving the TIR1 expression.

Compared with its F74A variant, the wild-type OsTIR1 performed poorly in *Y. lipolytica*, showing serious leaky degradation of mIAA7-tagged GFP in the absence of exogenous NAA addition. Auxin-independent leaky protein degradation with the wild-type OsTIR1 has been widely reported in several host systems (Yesbolatova et al., 2020). This phenomenon is generally believed (Mendoza-Ochoa et al., 2019) to result from 1) an intrinsic low affinity between the wild-type OsTIR1 and its substrate (i.e., the degron-tagged protein) even in the absence of auxin (Tan et al., 2007), 2) small amounts of auxin in the culture media, or 3) low levels of endogenous auxin in plants and yeast species such as *S. cerevisiae* (Rao et al., 2010) and *Y. lipolytica* (Gul Jan et al., 2019). The F74A mutation in OSTIR1 would enlarge its auxin-binding pocket to enable binding of 5-Ad-IAA or 5-Ph-IAA with a very high affinity, while its affinity for IAA is much lower (Nishimura et al., 2020; Zhang et al., 2022). As shown in Figure 4, when compared with 5-Ad-IAA, NAA induced much less protein degradation in combination with OsTIR1^F74A^ in *Y. lipolytica*.

In this study, β-carotene ketolase (Wps, encoded by *crtW*) was chosen as a target enzyme to illustrate the degron application in regulating metabolic pathways. This enzyme catalyzes the conversion of β-carotene to canthaxanthin (Supplementary Figure S3). By tagging the enzyme with the mIAA7 degron, we demonstrated that this enzyme could be degraded upon addition of Cu^2+^ and 5-Ad-IAA, and resulted in lower canthaxanthin production (Figure 5). The AID system enables post-translational regulation of proteins/enzymes via conditional degradation, which is complementary to transcriptional regulations such as use of inducible/repressible promoters and CRISPR activation (CRISPRa) or interference (CRISPRi), and post-transcriptional regulations such as RNA interference (RNAi). The AID approach is especially useful when the targeted genes are essential (and hence cannot be knocked out) and/or cannot be regulated transcriptionally. The inducible nature of the AID system and its rapid induction kinetics make it a powerful molecular tool for exerting tight temporal regulation of metabolic networks. The AID approach does have one drawback which is the need to tag the target endogenous proteins with the degron sequence for degradation, which requires modifying the host genome. However, with the advances in CRISPR genome editing, this barrier can be readily overcome. To exemplify its applications in metabolic engineering and synthetic biology, one may apply the AID in metabolic perturbation to elucidate metabolic network regulations, to redirect metabolic fluxes by cutting off byproduct synthesis, to implement temporal control of metabolic reaction networks, or to alter growth patterns (e.g., decoupling growth from product formation) by triggering depletion of protein targets essential for cell proliferation at a desired time point during the culture cycle. To this end, utility of AID1 (wt OsTIR1 coupled with the AID* degron tag, and NAA as the auxin inducer) in metabolic engineering of *S. cerevisiae* was demonstrated by the Vickers’ group who showed that AID1-mediated degradation of farnesyl pyrophosphate synthase increased the geranyl pyrophosphate pool which was redirected towards monoterpene production, whereas depleting acetyl-CoA carboxylase enabled decoupling of growth and production (Lu et al., 2021). As an extension of the present study to improve carotenoid production in *Y. lipolytica*, one may consider attenuating ergosterol synthesis via auxin-inducible degradation of squalene synthase as the cells enter stationary phase, to direct more farnesyl pyrophosphate towards geranylgeranyl pyrophosphate and downstream carotenoid biosynthesis.

## 5 Conclusion

In this study we demonstrated that the mIAA7/OsTIR1^F74A^/5-Ad-IAA AID system is functional in the industrially important oleaginous yeast *Y. lipolytica*. Conversely, the mIAA7/OsTIR1/NAA system works poorly with considerable leaky auxin-independent protein degradation. Meanwhile, our Western blot analyses revealed some degrees of proteolytic cleavage within the mIAA7 degron sequence whether it was fused internally or to the carboxyl terminus of the target protein. Work is currently underway to resolve this issue by creating alternative protein scaffolds to stabilize the degron structure. Having established an effective conditional-degron system for *Y. lipolytica* will greatly expand the synthetic-biology toolbox for this important organism to develop novel and more advanced traits.

## Data Availability

The datasets presented in this study can be found in online repositories. The names of the repository/repositories and accession number(s) can be found in the article/Supplementary Material.
