# Supplementary material for: Conditional protein degradation in Yarrowia lipolytica using the auxin-inducible degron
Source: Front Bioeng Biotechnol. 2023 May 31;11:1188119. doi: 10.3389/fbioe.2023.1188119 (PMC10264656; doi:10.3389/fbioe.2023.1188119)
Supplement: Supplementary file 1 [file DataSheet1.PDF]

Supplementary Material

Conditional protein degradation in *Yarrowia lipolytica* using the auxin-inducible degron

Zhenlin Han[1], Jessica Maruwan[1], Yinjie Tang[2], Wei Wen Su[1*]

[1]Department of Molecular Biosciences and Bioengineering, University of Hawaii at Manoa, Honolulu, Hawaii 96822, USA

[2]Department of Energy, Environmental and Chemical Engineering, Washington University, Saint Louis, Missouri 63130, USA

*** Correspondence:** Wei Wen Su; wsu@hawaii.edu

Table S1: Primers used in this study

Figure S1: Plasmid maps of major vectors.

Figure S2. Organization of major gene expression cassettes used in this study.

Figure S3. A simplified biosynthesis pathway that links key steps in the mevalonate pathway to β-carotene and canthaxanthin production.

Table S1. Primers used in this study

Primer	Primer sequence (5'-3')
C1T1F	TTCCGAACAGAAGGAATGCACGTCGACGCTCTCCCAGAGAC CGGG
TINR	GGATCCCTGCGGTTAGTACTGCAAAA
TIGF	TTTGCAGTACTAACCGCAGGGATCCGTGAGCAAGGGCGAGG AGC
SGPR	ACTCACTTCCCCATCCACACTTCCGCTAGCTTACTTGTACAG CTCGTCCA
SFGR	CTTGTACAGCTCGTCCAT
IA7F	TCACGGCATGGACGAGCTGTACAAGGGCTTCTCTGAGACCG TGGAC
IA7LR	TGATTCCGAACAGAAGGAATGCACGTCGACTTAGGA GCTTGTCTTCTGCTG
A7WF	CCCAGCAGAAGACAAGCTCCTCTGCCCACGCTCTGCCCAA
PWH6R	CACTTCCCCATCCACACTTCTTAATGGTGATGGTGATGGTGG GCGGTGTCGCCCTTAGT
TWPF	GCACTTTTTGCAGTACTAACCGCAGTCTGCCCACGCTCTGCC CAAG
M2GF	TACTTCCAAACTCACTATCTCAATGGGATCCGTGAGCAAGG GCGAGGAGC
SFGN	AACGTGGGGA CAGGCCATGG AGCTAGCTTA CTTGTACAGC TCGTCCA
MT2F	CGAAGTTATGCTCTCAAGGGCATCGGTCGACTGAATTCCATT ATGTGTGTGTGTG
MT2R	CATTGAGATAGTGAGTTTGGAAG
MOSF	TACTTCCAAACTCACTATCTCAATGACCTACTTCCCCGAAGA GGTGG
MOSR	GGCAACGTGGGGACAGGCCATGGATTACAGGATCTTCACGA AGTTGG
F74AF	CAAGCCCCATGCTGCGGACTTCA
F74AR	TGAAGTCCGCAGCATGGGGCTTG
QACT1F	CGAGCGAATGCACAAGGA
QACT1R	GCGGTGATCTTGACCTTGATG
QTEF1F	TTCACTGAGTACCCCCCTCTTG

QTEF1R	GCAACGGTCTGTCGCATGT
Q74F1	GCCCGATACGAGACCATGAG
Q74R1	GATCACCTCCACGTTCAGCA
QSGF	AGCAGAAGAACGGCATCAA
QSGR	GGGTGTTCTGCTGGTAGTG

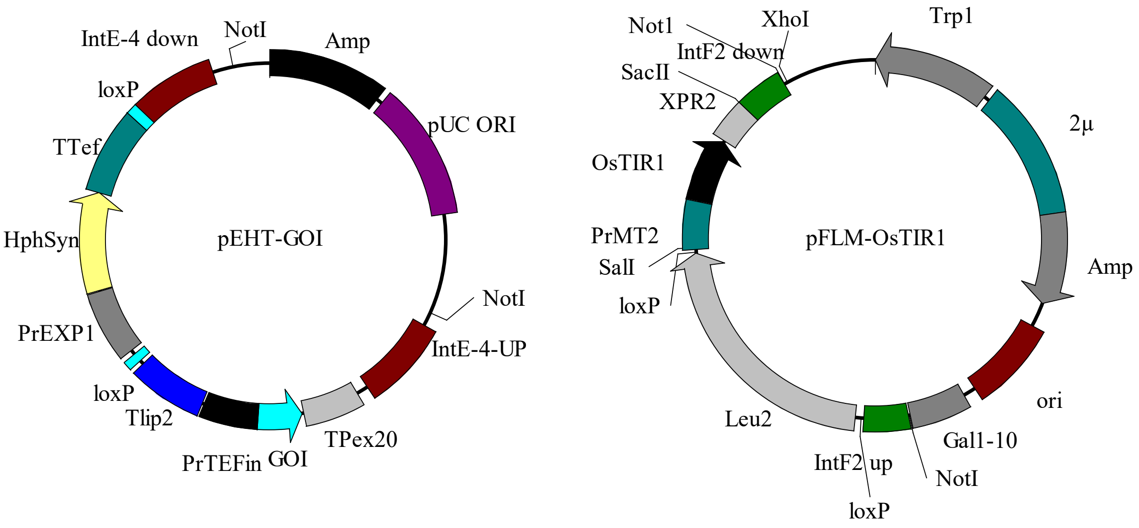

Figure S1. Plasmid maps of major vectors. A: pEHT vector for expressing GOI under the control of the TEF_{intron} promoter. B: pFLM vector for expressing wild-type OsTIR1 or $OsTIR1^{F74A}$ coding sequence driven by the MT2 promoter.

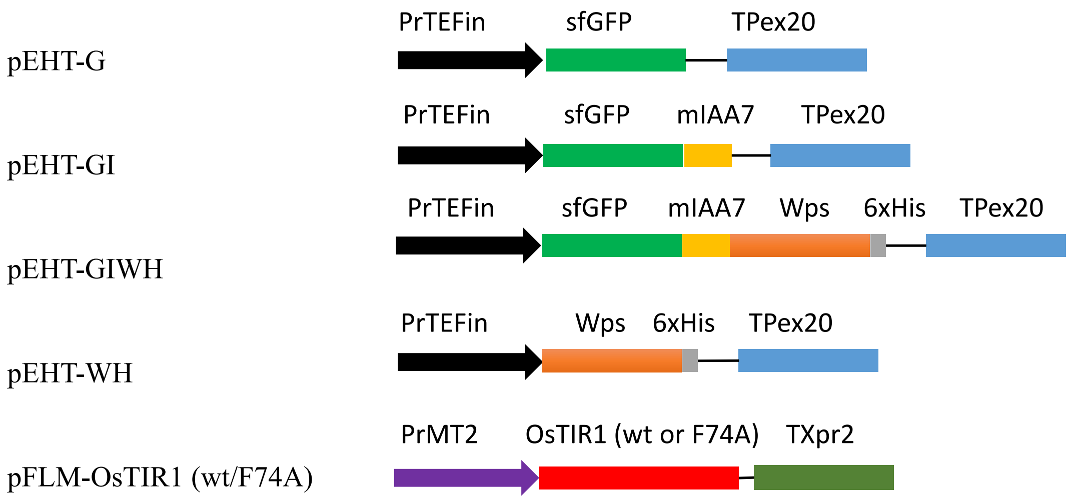

Figure S2. Organization of major gene expression cassettes used in this study. Expression of sfGFP, sfGFP-mIAA7, sfGFP-mIAA7-Wps-His_6, and Wps-His_6 was driven by the TEF_{intron} promoter and terminated with the Pex20 terminator. The expression of OsTIR1 (wt or F74A variant) was driven by the copper-inducible MT2 promoter and terminated with the Xpr2 terminator.

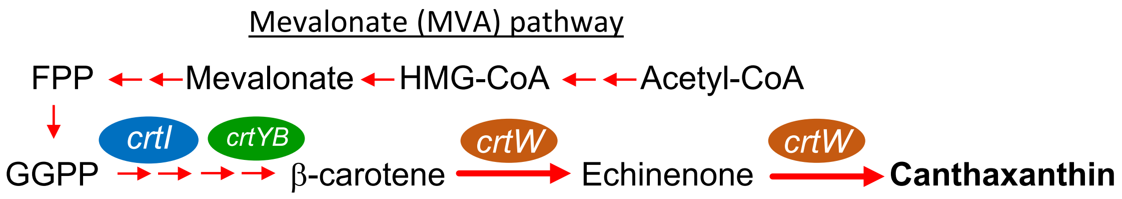

Figure S3. A simplified biosynthesis pathway that links key steps in the mevalonate pathway to β-carotene and canthaxanthin production. β-carotene is synthesized via the MVA pathway and by overexpressing heterologous *crtI* and *crtYB* genes (encoding phytoene desaturase and phytoene synthase/lycopene cyclase, respectively) in *Yarrowia lipolytica*. β-carotene ketolase (encoded by *crtW*) is over-expressed to convert β-carotene to canthaxanthin. FPP: farnesyl pyrophosphate; GGPP: geranylgeranyl pyrophosphate.
